# The comparison effects of intra-articular injection of Platelet Rich Plasma (PRP), Plasma Rich in Growth Factor (PRGF), Hyaluronic Acid (HA), and ozone in knee osteoarthritis; a one year randomized clinical trial

**DOI:** 10.1186/s12891-021-04017-x

**Published:** 2021-02-03

**Authors:** Seyed Ahmad Raeissadat, Parsa Ghazi Hosseini, Mohammad Hasan Bahrami, Reza Salman Roghani, Mohammad Fathi, Azadeh Gharooee Ahangar, Mahtab Darvish

**Affiliations:** 1 Clinical Development Research Center of Shahid Modarres Hospital, School of Medicine, Shahid Beheshti University of Medical Sciences, Tehran, Iran; 2 Physical Medicine and Rehabilitation Research Center, Shahid Beheshti University of Medical Sciences, Tehran, Iran; 3 Shohada-e-Tajrish Hospital, School of Medicine, Shahid Beheshti University of Medical Sciences, Tehran, Iran; 4 Department of Neurobiology, Care Sciences and Society, Karolinska Institute, Stockholm, Sweden; 5 Critical Care Fellowship, Department of Anesthesiology, Shahid Modarres Hospital, Shahid Beheshti University of Medical Sciences, Tehran, Iran; 6 Critical Care Quality Improvement Research Center, Shahid Beheshti University of Medical Sciences, Tehran, Iran

**Keywords:** Platelet rich plasma, Plasma rich in growth factor, Hyaluronic acid, Ozone, Knee osteoarthritis

## Abstract

**Background:**

Our study compare the short and long-term efficacy of the intra articular injections (IAIs) of hyaluronic acid (HA), platelet-rich plasma (PRP), plasma rich in growth factors (PRGF), and ozone in patients with knee osteoarthritis (OA).

**Methods:**

In this randomized clinical trial, 238 patients with mild to moderate knee OA were randomized into 4 groups of IAIs: HA (3 doses weekly), PRP (2 doses with 3 weeks interval), PRGF (2 doses with 3 weeks interval), and Ozone (3 doses weekly). Our outcome measures were the mean changes from baseline (immediately from the first injections) until 2,6, and 12 months post intervention in scores of visual analog scale (VAS), Western Ontario and McMaster Universities Osteoarthritis Index (WOMAC), and Lequesne index.

**Results:**

A total of 200 patients enrolled in the final analysis. The mean age of patients was 56.9 ± 6.3 years, and 69.5% were women. In 2 months follow up, significant improvement of pain, stiffness, and function were seen in all groups compared to the baseline, but the ozone group had the best results (*P* < 0.05). In 6 month follow up HA, PRP, and PRGF groups demonstrated better therapeutic effects in all scores in comparison with ozone (*P* < 0.05). At the end of the 12th month, only PRGF and PRP groups had better results versus HA and ozone groups in all scores (P < 0.05). Despite the fact that ozone showed better early results, its effects begin to wear off earlier than other products and ultimately disappear in 12 months.

**Conclusions:**

Ozone injection had rapid effects and better short-term results after 2 months, but its therapeutic effects did not persist after 6 months and at the 6-month follow up, PRP,PRGF and HA were superior to ozone. Only patients in PRP and PRGF groups improved symptoms persisted for 12 months. Therefore, these products could be the preferable choices for long-term management.

**Trial registration:**

Registered in the Iranian Center of Clinical Trials (www.irct.ir) in 11/11/2017 with the following code: IRCT2017082013442N17.

## Background

Knee osteoarthritis (OA) as a common progressive degenerative condition is one of the most important leading causes of disability and relative dependence [1]. Loss of jobs, early retirement, and arthroplasty are among the detrimental effects of this disease on individual quality of life and the disease burden on societies [2]. Worldwide prevalence of symptomatic knee OA has estimated 3.8% [3]. It affects more than 20% of over 45-year-old population [4]. Radiologic evidence suggestive of knee OA is seen in approximately 43% of the 50–60 year-old Iranians [5].

A multiplicity of treatments has been suggested for this disease; some of which include patient education, medication, exercise prescription, conventional and novel physical agent modalities such as laser therapy, and surgical management [6]. The current therapeutic options available for knee OA are not robustly effective and satisfactory for patients and pain has been complained of by at least 40% of those cases who underwent surgical arthroplasty [4]. Meanwhile, there is no a single well-known or approved remedy that can stop the progress of knee OA [5]. Therefore, in the last two decades, a large body of work has been performed to develop non-operative or minimally invasive interventions to alleviate OA symptoms or slow down OA progression. However, no consensus has been reached yet regarding the standard management strategies [7–11]. Among the minimally invasive methods recommended for knee OA management is intra-articular injections for which a large array of products have been used such as corticosteroids, dextrose, hyaluronic acid (HA), plasma derivatives including platelet-rich plasma (PRP) and plasma rich in growth factors (PRGF), and ozone [12, 13]. Although the intra-articular injections of corticosteroids have been shown to be effective, but in some situations these products may be less favored because of their short-term activity and adverse effects [14].

HA is a natural glycosaminoglycan found in the joints and provides the basis for synovial fluid viscoelastic characteristics [15]. Since during the knee OA the degradation of synovial fluid hyaluronate occurs, therefore it has been assumed that the intra-articular injection of HA could ameliorate the functional impairment and knee joint pain. In this regard, HA has been considered as a pharmacologic option and was approved by the FDA for knee OA in 1997 and recommended as an effective treatment for knee OA in the guideline of the American College of Rheumatology in 2000 [16, 17]. However, due to the controversial results, there is no agreement regarding the management of knee OA with intra-articular HA injections. Despite that some clinical guidelines do not recommend the use of HA for knee OA management, mainly because of less efficiency, but it is still used as a safe with minimal side-effects alternative [18, 19]. Furthermore, some other guidelines and several recent meta-analyzes support the use of viscosupplements in the management of knee OA [8, 20–22].

The autologous PRP is another biological product has gained more attention in the treatment of patients with knee OA in recent years. Several studies have been conducted worldwide supporting the use of PRP injection as an effective method for knee OA [23]. Numerous studies have used PRP in different settings and the obtained results show that PRP could serve as antinociceptive and induce cell proliferation [24]. It has also been shown that the intra-articular injection of PRP modulates joint environment, promote chondrogenesis and inhibits the destruction of knee joint probably by reducing the production of pro-inflammatory mediators [25]. The therapeutic effects of the PRP might be also explained by the supra-physiologic concentrations of biological molecules and growth factors exist in in the granules of the platelets which could potentially reverse the catabolic environment in OA, balancing the homeostasis of the joint, and subsequently stimulate the repair of damaged cartilage [23, 26]. However, similar to what mentioned about the HA, there is discrepancy in the literature concerning the widespread use of intra-articular PRP to treat knee OA in clinical practice [27]. Such controversies have been attributed to the post injection release of growth factors from platelets. It is possible that, for some reasons, a percentage of growth factors are not released post injection, and leads to the low treatment response. To circumvent this impediment, biologic activators compatible with body have been used to stimulate the platelets to release their granular content which resulted in the creation of PRGF [28]. In fact, PRGF is the final product of PRP, without leukocytes and inflammatory cytokines and only contains a specific amount of cytokines and growth factors. This makes PRGF more effective and lessens its side effects such as pain and swelling compared to PRP [27].

More recently, there has also been growing interests towards the use of ozone as a safe option in managing knee OA patients. There are several advantages associated with the ozone therapy including the ease of administration and low cost of application. Intra-articular injection of ozone is considered as one of the effective treatments in improving the symptoms of knee OA [29]. It has been proven that the intra-articular injection of ozone, as a liquid form (mixture of oxygen and ozone), could improve mild to moderate knee OA [29]. Mechanistically, the mixture of oxygen and ozone can improve tissue oxygenation, accelerate the generation of reactive oxygen species and thereby could decrease the release of proinflammatory cytokines, which consequently counteracts with the activation and recruitment of leukocytes and other types of cells into the inflammatory site and thus relives the symptoms of knee OA [30]. Although it has been shown that ozone therapy could exert short-term effects, but inconsistent results have been reported regarding its long-term effects [31].

Based on the mentioned notes, and to the best of our knowledge there is still lack of general consensus on the choice and priority of the intra-articular HA, PRP, PRGF, and ozone injections in the management of knee OA. Accordingly, previous studies evaluated the inter-individual difference of HA, PRP, PRGF, and ozone injections have not achieved the same results. Therefore, in the present study, we aimed to comparatively examine the short and long-term effectiveness (2 months and 12 months after interventions, respectively) of the intra-articular injections of HA, PRP, PRGF, and ozone in knee OA improvement.

## Methods

### Study design

The current study was a randomized clinical trial that was performed from December 2017 until February 2019 with the aim of comparing the long-term effects of 4 intra-articular injections of HA, PRP, PRGF, and ozone on the symptoms of patients suffering from mild to moderate osteoarthritis who referred to the physical medicine and rehabilitation clinic of Shahid Modarres hospital in Tehran.

### Inclusion and exclusion criteria

The cases were the consecutive out-patients aged 50–75 years referred to the Physical Medicine and Rehabilitation clinic of Modarres Hospital in Tehran who suffered from the knee pain and had symptoms for longer than 3 months. After being examined, the patients who diagnosed with knee OA based the criteria of the American College of Rheumatology according to knee X-ray) were completely informed about the design, methodology and voluntary nature of this research and enrolled in the study with their consent. Indeed, the definition and diagnosis of OA was based on the ACR criteria and the classification of OA patients was performed based on the Kellgren and Lawrence grading system [32]. Patients diagnosed with knee OA (grade 2 or 3. Exclusion criteria were: having systemic disease such as diabetes mellitus, immunodeficiency, collagen vascular disease, history of malignancy, infection or active wound in the knee, autoimmune diseases, disorders affecting platelets, use of NSAIDs 2 days prior to injection, uses anticoagulant or anti-platelet 10 days before injection, steroid knee injection 3 weeks before the procedure, systemic steroid injection in previous 2 weeks, hemoglobin< 12 mg/dl or platelet< 150,000/μl, history of severe knee trauma, history of vasovagal shock, pregnancy, lactation, genu-valgum or genu-varum more than 20 degrees, history of allergy to egg protein, chicken proteins or chicken feather or hypersensitivity to hyaluronate, treatment with ACE inhibitors or G6PD deficiency.

### Ethical considerations

All goals of the study, expected results, and follow up steps were explained to the candidates and they were assured that all their information would remain private. Since the usual treatment for osteoarthritis is exercise and medication, this treatment was additionally used for all four groups [33, 34]. Accordingly, all the cases in the 4 intervention groups equally underwent routine exercise if there no contraindication was observed. Furthermore, we examined the medical history of the patients and the medications that had previously been prescribed for them. Based on the exclusion criteria, the previous medications were discontinued for the all cases of all groups and the acetaminophen was the only choice where participant(s) complained of pain. In the critical situations during the study NSAIDs were prescribed for shorter durations. Written consent was acquired from candidates and they were allowed to leave the study any time that they wanted. This study has been approved by Shahid Beheshti University of Medical Sciences’ ethical community with the following code: IR.SBMU.MSP.REC.1396.230 and has been registered in the Iranian Center of Clinical Trials (www.irct.ir) with the following code: IRCT2017082013442N17.

### Randomization and enrolment

**Fig. 1:**
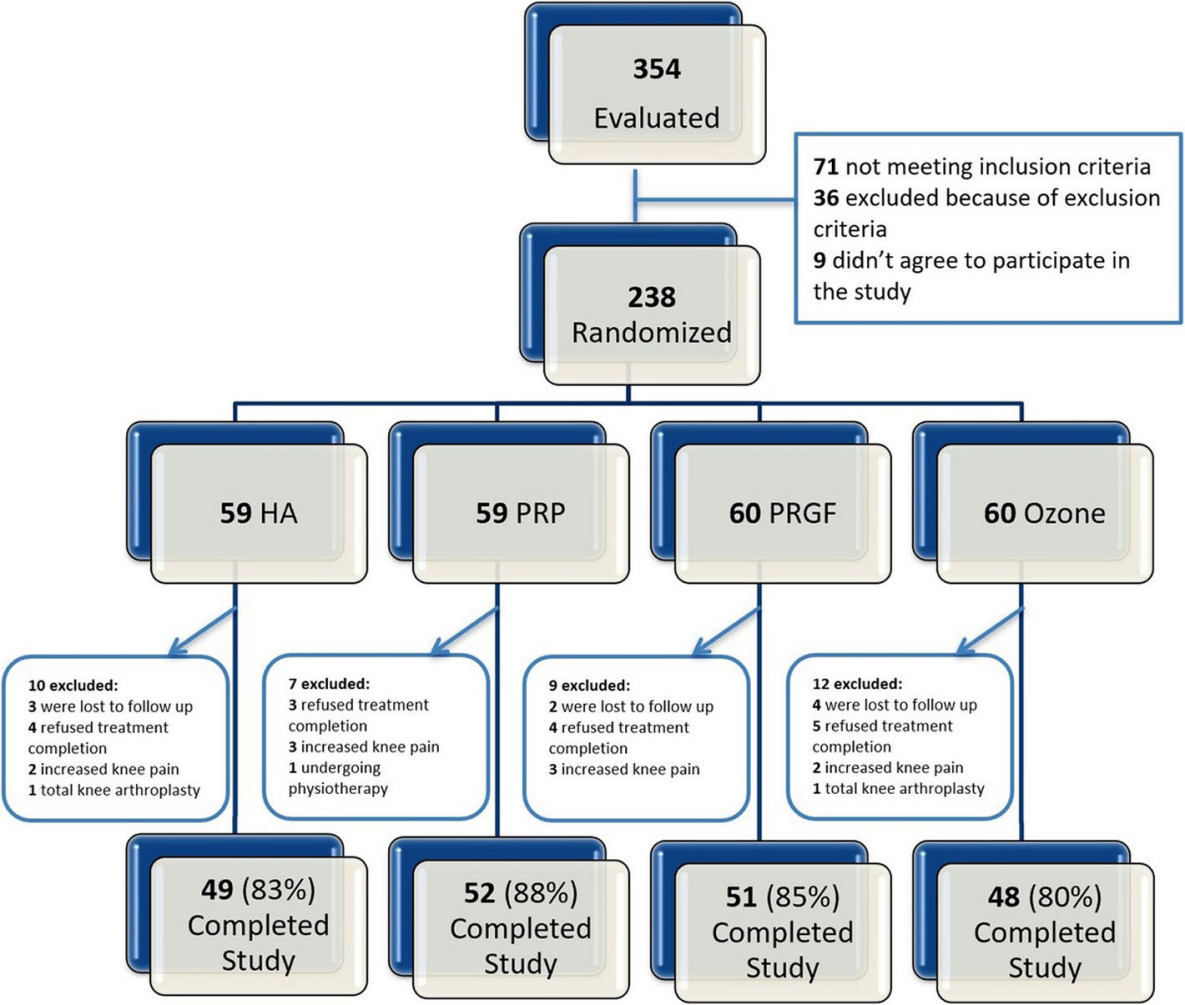
Enrollment and allocation diagram

Overall, 354 patients were evaluated, which included history, physical examination, lab tests including complete blood count (CBC), C-reactive protein (CRP), and erythrocyte sedimentation rate (ESR), anteroposterior (AP) and lateral standing knee X-rays, and assessment of medications and supplements received by the candidates. At the end, 238 subjects were allocated through permuted block randomization method by the use of random allocation software into 4 groups of HA, PRP, PRGF, and ozone, where they distributed in 15 blocks with 16 cases in each block. None of the participants in the study were aware of randomization process and sealed envelopes were used to conceal the randomization assignments. It has to be mentioned that, the trial was parallel-group in nature with 1:1 allocation ratio. The recruitment and randomization were done by a resident assistant in physical medicine and rehabilitation who was not blinded to subject allocations. All study subjects were visited and interviewed at clinic 2, 6, and 12 months after interventions by another resident assistant who was blinded to subject allocations. Finally, 200 subjects remained in the study (Fig. 1).

### Interventions

All the injections for the all groups were prepared by an experienced nurse and administered by a blind clinician specialized in physical medicine and rehabilitation. The intra-articular knee injections were done through the lateral mid-patellar approach, while the knee was in the extension position. The syringes were covered with a trial label to mask the contents from all and had identical appearances, thus the administering clinician were blinded to the interventions. The number of injections and the time intervals between different injections differed in many studies; however, both the injection numbers and time points in this study were based on our previous experiences. The description of injections and time intervals between the injections in this research was as follows: HA (3 doses weekly), PRP (2 doses with 3 weeks interval), PRGF (2 doses with 3 weeks interval), and Ozone (3 doses weekly). In more details, In the HA group, the product with the trademark of Hyalgan was used. Hyalgan is a synthetic hyaluronic acid made by Italy’s Fidia Farmaceutici S.P.A, Abano Terme and is a viscous solution containing molecules with the molecular weight between 500 to 730 k Daltons that has been buffered in physiologic sodium chloride. The PH of this product is 6.8–7.5. The injection was performed in a sterile environment using a G20 needle and the classic (medial and lateral infrapatellar) approach. The patient was asked to actively perform knee flexion and extensions. The second and third injections were performed weekly under similar conditions.

In the PRP group, for PRP preparation, a Royagen kit (made by Arya Mabna Tashkis Co. SN: 312569) was used [35]. In this process 35 cc of blood was taken from the ante cubital vein using a G18 needle. Afterwards, 5 cc of acid citrate dextrose was added as anticoagulant. The blood sample was centrifuged for 15 min at 1600 rpm, which resulted in three separate layers. The lower layer being RBC precipitate, the middle layer WBCs and the top layer plasma. Plasma alongside the buffy coat layer was removed to be centrifuged for 7 min at 3500 rpm. In the final stage, 2 cc of plasma remains in each tube, which has achieved a platelet concentration of almost 4–6 times in PRP group. However, in PRGF group, for each 2 cc of PRP, 1.5 cc of Royagen platelet activating factor (epinephrine, and 25 mM calcium chloride) was added and turned upside-down 4 times to mix them up and achieve the final concentration of 5 times normal. The final PRP solution was put inside a Royagen warm water device (40 ± 1 °C) for 20–30 min so that the platelets would release their factors. Finally, two phases are created; a liquid part, which is actually PRGF at the bottom, and a solid part on top, which is platelet remnants. The PRGF can be acquired using a syringe or centrifuging the solution at 4000 rpm for 4 min until the platelet residue sticks to the bottom of the test tube. To facilitate the acquisition of the PRGF, the second method is recommended. The injection of PRP and PRGF was performed using a G22 needle and the classic approach. After 15–20 min of rest, the patient was asked to actively flex and extend their knee. The second injection was performed after 3 weeks under similar circumstances [12].

In the ozone group, under sterile conditions and using a G22 needle, 10 cc of ozone-oxygen with a predetermined concentration of 30 micrograms per cc was injected using the classic approach. The second and third injections were performed weekly under similar circumstances. In this group, we used Ozonibaric P ozone generator (made by Sedecal, Spain) [29].

All patients were recommended to have relative rest for 24–48 h and limited weight bearing on the injected knee was performed. They were also recommended to use cold compression 3 times a day for 10 min up to 72 h. Patients were allowed to paracetamol (without codeine) every 8 h. If the pain persisted, every 4 h, but use of any other form of analgesic or anti-inflammatory such as NSAIDs, steroids, or drugs that affect platelets was not permitted until 5 days after injection. Exercise therapy was prescribed for all candidates, which was explained to all candidates by a physical medicine and rehabilitation resident before injection. The exercise therapy protocol used was multi angle isometric exercises of muscles surrounding the knee (quadriceps femoris, thigh abductors and adductors) as well as stretching of hamstrings 3 times a day, 10 times for each move for 10 s. Patients were encouraged to gradually change to closed chain isotonic exercises after a month.

### Measurement parameters

In order to assess the effects of this study three tools were used; the Visual Analogue Scale (VAS), Western Ontario and McMaster Universities Arthritis Index (WOMAC), and Lequesne algofunctional index, which have been widely used in studies. The WOMAC questionnaire contains 24 items: 5 items for pain, 2 items for joint stiffness, and 17 items for functional limitations. Each item is scored from 0 to 5 on a Likert scale. A lower score on this scale implies less pain and more function. The Farsi translation of this questionnaire has been evaluated for validity [3]. The Lequesne questionnaire has 11 items: 5 items for pain, 2 items for maximal walking rate, and 4 items for activities of daily life (ADL). A lower score is also associated with less pain and better function. This questionnaire also has validated in Farsi [3]. The VAS scale is a subjective scale and is used to quantitatively assess pain (0–10, 0 = No pain, 10 = Severe pain).

All assessments were made using VAS, WOMAC, and Lequesne algofuctional index at the beginning of the study as well as in the 2nd, 6th, and 12th month after intervention by a physical medicine and rehabilitation resident who was unaware of the injected product for each patient. It is worthy to note that, the questionaries were printed and given to the participants in the hard copy form and were filled by the help of a blind assessor (specialized in physical medicine and rehabilitation). In addition, post injection pain was assessed immediately after each injection within 5 min for all injections. During the final assessment at 12 months, the patients’ satisfaction of treatment was asked using a visual scale grading from “very much” to “very little”. Furthermore, after each injection the patients were followed up by phone calls for 1 week for possible post injection adverse events including pain, heaviness, stiffness and mild effusion. Any signs of infection (redness, severe pain, severe inflammation) in the injection site has been considered as a serious complication. If they had signs of such events, they were asked to be visited and physical examinations were performed.

### Sample size calculation

The calculated sample size was 50 in each group considering the results from earlier study^7^ with a regard to significant mean difference in decreased scores of WOMAC and Lequesne, the equation for calculating the sample size to compare two means, the test power of 80% at the significance level of 0.5.

### Statistical analysis

The gathered data were analyzed by a medical statistics expert who was unaware of the groupings. The software used for data analysis was STATA 14 and the figures were provided by Prism version 5. Continuous demographic variables were expressed as mean ± SD, whilst categorical variables were expressed as percentages of the total group. Modified intention to treat (overall success) analysis was performed on all participants randomized into the groups. Only patients with missing data were excluded from statistical analysis and for available cases (AC), the generalized estimating equations (GEE) method was used for longitudinal data analysis. In the GEE analysis, two parts including time and group and the time-group interaction are considered and the correlation matrix was considered exchangeable for each outcome. Interventional effects were adjusted by the baseline level and patients’ demographics. *P* value < 0.05 was considered as significant throughout the study.

## Results

In this randomized clinical trial, 200 patients with mild to moderate knee OA were studied. The aim of the study was to assess and compare the results of the different treatment groups of HA, PRP, PRGF, and ozone using WOMAC, VAS, and Lequesne at the beginning as well as 2, 6, and 12 months after the intervention. Patients were randomly categorized into each group of intra-articular injection. The group allocation was as follows: 52 patients in PRP, 51 in PRGF, 49 in HA, and 48 in the ozone group. Demographic data and patient history has been shown in Table 1, in which no significant difference was observed between the four groups (*P* > 0.05).

**Table 1:** Baseline characteristics of study participants

	Total (*n* = 200)	PRP (*n* = 52)	PRGF (*n* = 51)	HA (*n* = 49)	Ozone (*n* = 48)
**Baseline characteristics**					
Age (yr),Mean ± SD	56.9 ± 6.3	56.09 ± 6.0	56.07 ± 6.3	57.91 ± 6.7	57.60 ± 6.1
Sex (Male/Female)	61/139	13/39	14/37	12/37	12/36
BMI (kg/m2), Mean ± SD	28.24 ± 2.8	27.41 ± 2.6	27.50 ± 2.1	27.46 ± 2.2	27.01 ± 1.9
Duration of pain (yr), Mean ± SD	4.41 ± 2.2	4.44 ± 2.3	4.9 ± 2.7	3.86 ± 1.6	4.42 ± 2.1
Side of injection (left/right)	93/107	22/30	18/33	28/21	25/23
Degree of osteoarthritis (2/3)	108/92	26/26	28/23	27/22	27/21
History physiotherapy, n(%)	119 (59.5)	29 (55.8)	36 (70.6)	26 (53.1)	28 (42.3)
History injection, n(%)	92 (46.0)	22 (44.3)	25 (49.0)	24 (49.0)	21 (58.3)
Pain during injection, Mean ± SD	2.43 ± 2.0	2.80 ± 2.2	3.07 ± 2.6	1.81 ± 1.3	1.95 ± 1.18
**Outcome measures**					
VAS, Mean ± SD	8.03 ± 1.2	7.92 ± 1.0	7.90 ± 1.3	8.22 ± 1.1	8.10 ± 1.0
WOMAC, Mean ± SD					
Pain	9.54 ± 1.6	9.69 ± 1.3	9.72 ± 1.7	9.44 ± 1.6	9.29 ± 1.8
Function	30.68 ± 7.3	30.19 ± 6.4	30.54 ± 7.6	31.02 ± 8.8	31.00 ± 6.1
Stiffness	2.73 ± 1.3	2.84 ± 1.1	2.84 ± 1.6	2.71 ± 1.1	2.50 ± 1.1
Total	42.85 ± 9.2	42.73 ± 7.7	43.11 ± 9.6	42.75 ± 11.1	42.79 ± 8.2
LEQ, Mean ± SD					
Pain	5.31 ± 1.0	5.17 ± 1.0	5.13 ± 1.1	5.55 ± 0.9	5.41 ± 1.0
Walk	1.65 ± 0.8	1.65 ± 0.6	1.66 ± 0.8	1.71 ± 0.9	1.56 ± 0.7
ADL	5.71 ± 0.7	5.75 ± 0.6	5.71 ± 0.7	5.70 ± 0.8	5.67 ± 0.7
Total	12.65 ± 2.0	12.58 ± 1.6	12.62 ± 2.1	12.76 ± 2.2	12.65 ± 2.0

Abbreviations: *SD* standard deviation; *PRGF* plasma rich in growth factor; *PRP* platelet-rich plasma; *HA* hyaluronic acid; *VAS* visual analog scale; *WOMAC* Western Ontario and McMaster Universities Osteoarthritis Index; *LEQ* Lequesne Index

**Fig. 2:**
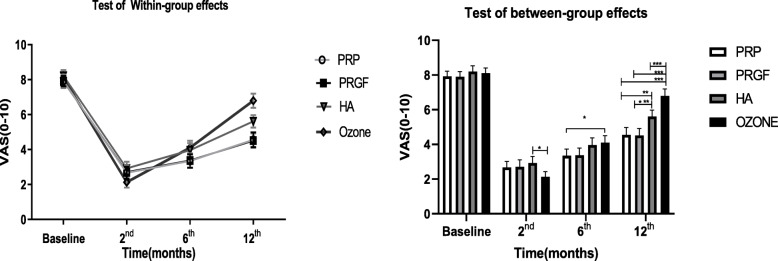
Bar chart of the VAS score within and between the groups at the beginning, and 2, 6 and 12 months of follow up

**Fig. 3:**
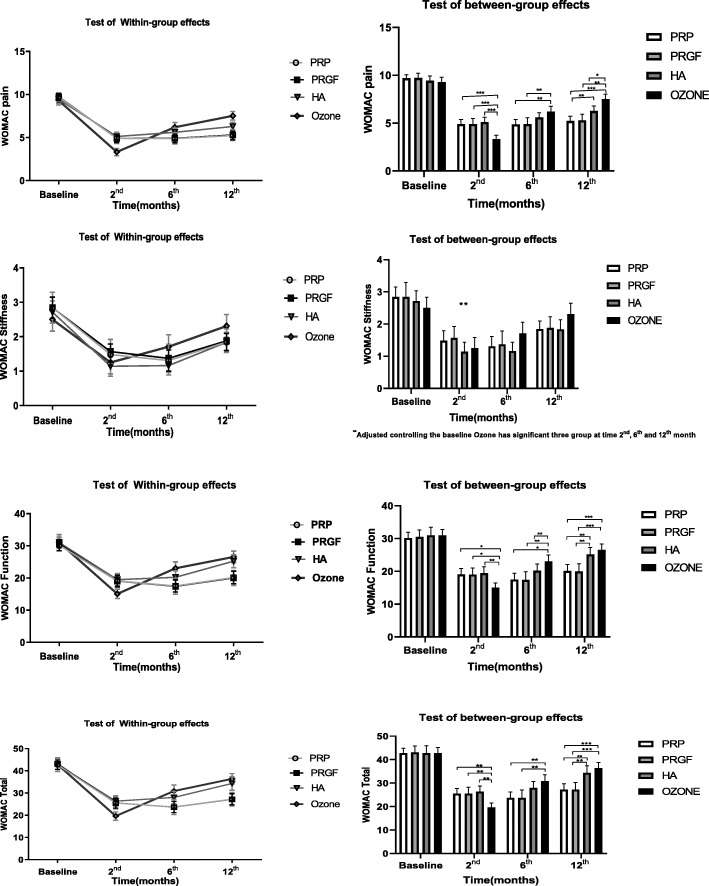
Bar chart of the WOMAC scores within and between the groups at the beginning, and 2, 6 and 12 months of follow up

**Fig. 4:**
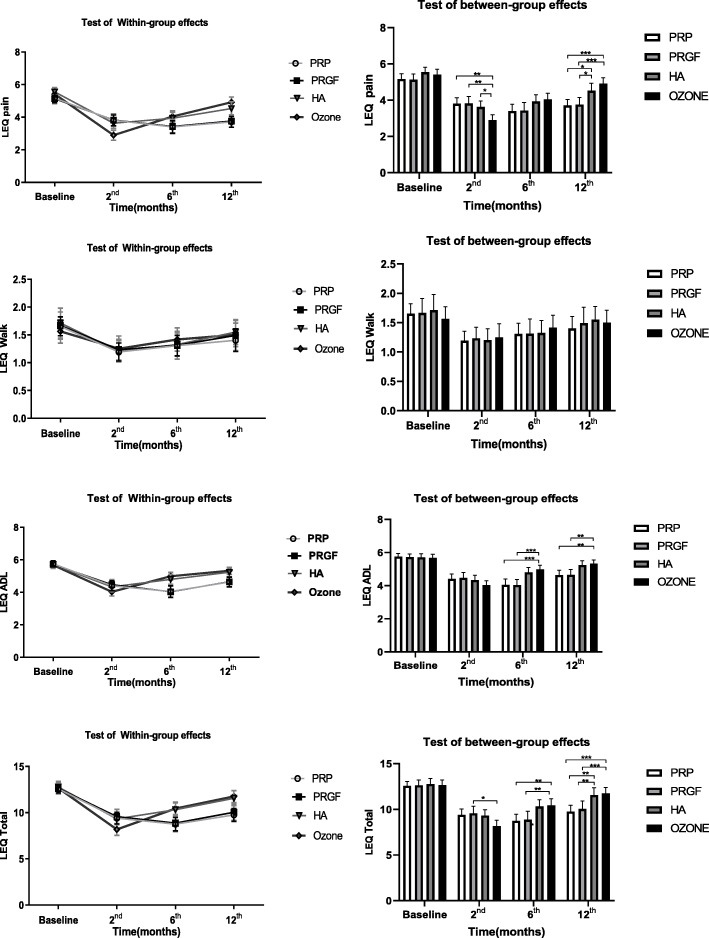
Bar chart of the LEQ scores within and between the groups at the beginning, and 2, 6 and 12 months of follow up

To compare the responses of the knee OA patients to the different treatment modalities, we performed intra and inter-group assays based on the data obtained by using WOMAC, VAS, and Lequesne scores at the beginning of the study as well as 2, 6, and 12 months after injections (Tables 2, 3, and Figs. 2, 3 and 4). The primary outcome measure was the pain relief and functional improvement based on the WOMAC score as well as the improvement in the Lequesne total score and sub-scores including pain, ADL and MWD. The secondary outcome measure was the patients’ consent and side effects related to the injections. Of note, we considered 30% reductions in WOMAC and VAS as worthwhile treatment effects.

**Table 2:** Mean difference within-groups at 2, 6 and 12 months follow up (available case analysis by GEE)

	Test of Within-group effect) mean change from baseline)				Between-group	
	PRP(n = 52)	PRGF (n = 51)	HA(n = 49)	Ozone (n = 48)		
**Outcomes**	MD^a^(95%CI)	MD^a^(95%CI)	MD^a^(95%CI)	MD^a^(95% CI)	P value^#^	P value^##^
**WOMAC**						
**Pain** T2	−4.8 (−5.2,-4.3)^***^	−4.8(− 5.4,-4.2)^***^	− 4.3(− 4.6,-3.9)^***^	−5.9(−6.4,-5.5)^***^	< 0.001	< 0.001
T6	− 4.8(− 5.2,-4.3)^***^	−4.8(− 5.4,-4.2)^***^	−3.8(− 4.1,-3.4)^***^	−3.1(− 3.5,-2.6)^***^	< 0.001	0.003
T12	−4.4(− 4.9,-4.0)^***^	−4.4(− 4.9,-3.8)^***^	−3.1(− 3.5,-2.8)^***^	− 1.7(− 2.2,− 1.3)^***^	< 0.001	< 0.001
FRACTION^b^	45.52% (40.1,50.9)	45.37 (39.1,51.6)	33.68% (29.4,37.9)	21.72 (17.5,25.8)		
**Stiff** T2	− 1.3(− 1.6,-1.0)^***^	-1.3(− 1.6,-0.88)^***^	−1.5(− 1.8,-1.3)^***^	−1.2(− 1.4,-1.0)^***^	0.93	0.23
T6	−1.5(− 1.8,-1.2)^***^	−1.5(− 1.8,-1.0)^***^	−1.5(− 1.7,-1.3)^***^	−0.8(− 1.0,-0.5)^***^	0.002	0.16
T12	− 1.0(− 1.3,-0.7)^***^	−0.96(− 1.3,-0.6)^***^	−0.8(− 1.1,-0.6)^***^	−0.2(− 0.4,0.03)^***^	< 0.001	0.09
FRACTION^b^	40.09% (29.7,50.4)	38.15% (29.1,47.2)	38.71% (29.9,47.4)	30.86% (19.7,41.9)		
**Fun** T2	− 11.1(− 12.6,-9.6)^***^	− 11.4(− 13.2,-9.7)^***^	−11.5(− 12.8,-10.2)^***^	−15.9(− 17.0,-14.8)^***^	0.008	0.002
T6	−12.6(− 14.1,-11.1)^***^	− 13.1(− 14.-11.4)^***^	−10.8(− 12.1,-9.4)^***^	−8.0(− 9.1,-6.8)^***^	< 0.001	< 0.001
T12	− 10.0(− 11.0,-8.8)^***^	− 10.5(− 12.2,-8.8)^***^	−5.8(− 7.1,− 4.4)^***^	-4.4(− 5.5,-3.3)	< 0.001	< 0.001
FRACTION^b^	33.99% (28.5,39.4)	37.41% (31.9,42.9)	21.22% (17.2,25.2)	14.99% (11.8,18.1)		
**Tota**l T2	−17.2(− 19.1,-15.3)^***^	− 17.5(− 19.9,-15.3)^***^	−16.4(− 18.1,-14.6)^***^	−23.1(− 24.5,-21.7)^***^	< 0.001	< 0.001
T6	−19.0(− 20.9,-17.1)^***^	− 19.4(− 21.7,-17.1)^***^	− 14.7(− 16.4,-12.9)^***^	−11.9(− 13.2,-10.5)^***^	< 0.001	0.001
T12	−15.5(− 17.4,-13.6)^***^	−15.9(− 18.2,-13.6)^***^	−8.4(− 10.1,-6.7)^***^	−6.4(− 7.7,-5.0)^***^	< 0.001	< 0.001
FRACTION^b^	36.50% (31.2,41.7)	38.5% (32.9,44.09)	23.08% (19.8,26.4)	15.5% (12.4,18.6)		
**LEQ**						
**Pain** T2	− 1.3(− 1.7,-1.0)^***^	− 1.3(− 1.6,-0.9)^***^	−1.9(− 2.2,-1.6)^***^	− 2.5(− 2.8,-2.2)^***^	< 0.001	< 0.001
T6	− 1.7(− 2.1,-1.4)^***^	− 1.7(− 2.0,-1.3)^***^	−1.6(− 1.9,− 1.3)^***^	-1.3(− 1.6,-1.10)^***^	0.089	0.08
T12	−1.4(− 1.8,-1.1)^***^	−1.3(− 1.7,− 1.0)^***^	-1.0(− 1.3,-0.7)^***^	− 0.5(− 0.7,-0.2)^***^	< 0.001	< 0.001
FRACTION^b^	27.37% (21.4,33.3)	27.96% (21.7,34.2)	25.77% (20.3,31.2)	16.11% (11.9,20.2)		
**Walk** T2	− 0.46(− 0.6,-0.3)^***^	− 0.4(− 0.6,-0.2)^***^	−0.5(− 0.7,− 0.3)^***^	−0.3(− 0.4,-0.2)^***^	0.34	0.97
T6	−0.3(− 0.5,-0.2)^***^	-0.3(− 0.5,-0.2)^***^	−0.4(− 0.5,-0.2)^***^	−0.14(− 0.3,-0.02)^*^	0.14	0.88
T12	−0.25(− 0.4,-0.07)^**^	−0.2(− 0.3,0.01)	−0.16(− 0.3,0.01)	−0.06(− 0.2,0.06)	0.13	0.84
FRACTION^b^	39.42% (28.6,50.2)	30.38% (19.5,41.2)	21.06% (11.4,30.7)	13.76% (4.8,22,7)		
**ADL** T2	− 1.3(− 1.6,-1.0)^***^	− 1.2(− 1.5,-0.9)^***^	−1.3(− 1.5,-1.2)^***^	−1.3(− 1.5,-1.2)^***^	0.077	0.19
T6	−1.7(− 2.0,-1.4)^***^	− 1.7(− 1.9,-1.4)^***^	−0.9(− 1.1,-0.7)^***^	−0.9(− 1.1,-0.7)^***^	< 0.001	< 0.001
T12	−1.1(− 1.4,-0.8)^***^	−1.1(− 1.3,-0.8)^***^	−0.4(− 0.6,-0.3)^***^	−0.4(− 0.6,-0.3)^***^	< 0.001	< 0.001
FRACTION^b^	21.7% (17.2,26.2)	22.2% (17.7,26.7)	10.89% (8.5,13.3)	7.07% (5.3,8.8)		
**Total** T2	− 3.2(− 3.7,-2.6)^***^	− 3.0(− 3.7,-2.4)^***^	− 3.4(− 3.8,-2.9)^***^	− 4.5(− 4.8,-4.1)^***^	< 0.001	0.021
T6	− 3.8(− 4.4,-3.2)^***^	−3.7(− 4.4,-3.1)^***^	− 2.4(− 2.9,-1.9)^***^	−2.2(− 2.5,-1.8)^***^	< 0.001	0.006
T12	−2.8(− 3.4,-2.3)^***^	−2.6(− 3.2,-1.9)^***^	− 1.2(− 1.6,-0.7)^***^	− 0.9(− 1.2,-0.5)^***^	< 0.001	< 0.001
FRACTION^b^	23.52% (19.0,28.0)	22.58% (17.7,27.4)	13.37% (10.3,16.5)	11.03% (8.6,13.4)		
**VAS (1–10)**						
T2	−5.2(− 5.6,-4.8)^***^	−5.2(− 5.6,-4.8)^***^	−5.3(− 5.6,-4.9)^***^	−5.9(− 6.3,-5.6)^***^	0.008	0.022
T6	−4.6(− 4.9,-4.2)^***^	−4.5(− 4.9,-4.1)^***^	−4.2(− 4.6,-3.9)^***^	−4.0(− 4.3,-3.7)^***^	0.02	0.013
T12	−3.3(− 3.7,-2.9)^***^	−3.4(− 3.7,-3.0)^***^	−2.6(− 2.9,-2.3)^***^	−1.3(− 1.6,-1.0)^***^	< 0.001	< 0.001
FRACTION^b^	42.37% (37.2,47.5)	42.38% (37.3,47.5)	31.59% (27.6,22.1)	18.69% (15.2,22.1)		

MD, mean difference; CI, confidences interval; T2, 2nd month post injection; T6, 6th month post injection; T12, 12th month post injection;

^a^ 2nd month−baseline; ^b^(|Baseline− 12th month|/Baseline)*100; *Within-group effects *p* < 0.05; **Within-group effects *p* < 0.01; **Within-group effects *p* < 0.001

^#^ Adjusted generalized estimating equations model after controlling the baseline Outcome, sex, age, BMI;

^##^ crude repeated measures AVOVA

**Table 3:** Mean difference between-group at 2, 6 and 12 months follow up (Adjusted analysis by GEE model)

	Test of Between-group (mean change from reference group)					
	PRGF vs PRP	HA vs PRP	Ozone vs PRP	HA vs PRGF	Ozone vs PRGF	Ozone vs HA
**Outcomes**	MD^a^ (95%CI)	MD^a^ (95%CI)	MD^a^ (95%CI)	MD^a^ (95% CI)	MD^a^ (95% CI)	MD^a^ (95% CI)
**WOMAC**						
**Pain** T2	− 0.03(− 0.67,0.60)	0.46(− 0.18,1.10)	− 1.2(− 1.8,-0.54)^***^	0.49(− 0.15,1.14)	− 1.15(− 1.80,-0.50)^***^	−1.65(− 2.30,-0.99)^***^
T6	−0.01(− 0.65,0.62)	0.97 (0.32,1.61)^**^	1.70 (1.05,2.35)^***^	0.98 (0.33,1.63)^**^	1.71 (1.06,2.37)^***^	0.73 (0.07,1.38)^*^
T12	0.03(−0.60,0.66)	1.29 (0.65,1.94)^***^	2.69 (2.04,3.33)^***^	1.26 (0.62,1.91)^***^	2.66 (2.0,3.31)^***^	1.39 (0.73,2.04)^***^
**Stiff** T2	0.09(− 0.32,0.50)	−0.20(− 0.62,0.21)	0.11(− 0.30,0.54)	− 0.29(− 0.72,0.12)	0.02(− 0.40,0.45)	0.32 (− 0.10,0.75)
T6	0.06(− 0.34,0.48)	−0.01(− 0.43,0.40)	0.74 (0.32,1.17)^***^	−0.08(− 0.50,0.34)	0.67 (0.25,1.10)^**^	0.75 (0.32,1.18)^***^
T12	− 1.0(− 1.3,-0.7)	− 0.96(− 1.3,-0.6)	−0.8(− 1.1,-0.6)^***^	0.08(− 0.34,0.50)	0.77 (0.34,1.19)^***^	0.69 (0.25,1.12)^**^
**Fun** T2	0.09(− 0.32,0.50)	−0.20(− 0.62,0.21)	0.11(− 0.30,0.53)	−0.06(− 2.11,1.99)	−4.44(− 6.51,-2.38)^***^	−4.38(− 6.47,-2.30)^***^
T6	0.07(− 0.34,0.40)	− 0.01(− 0.43,0.40)	0.74 (0.32,1.17)^***^	2.32 (0.26,4.37)^*^	5.13 (3.07,7.20)^***^	2.81 (0.73,4.90)^**^
T12	0.04(− 0.37,0.45)	0.12(− 0.29,0.54)	0.81 (0.38,1.23)^***^	4.73 (2.67,6.78)^***^	6.09 (4.02,8.15)^***^	1.35(− 0.72,3.44)
**Tota**l T2	− 0.31(− 2.94,2.30)	0.88(− 1.76,3.53)	− 5.89(− 8.56,-3.23)^***^	1.20(− 1.46,3.86)	− 5.5(− 8.25,-2.90)^***^	−6.77(− 9.48,-4.07)^***^
T6	−0.41(− 3.03,2.20)	4.30 (1.65,6.95)^***^	7.12 (4.46,9.78)^***^	4.71 (2.05,7.37)^***^	7.53 (4.86,10.21)^***^	2.81 (0.11,5.52)^*^
T12	−0.40(−3.02,2.21)	7.09 (4.44,9.73)^***^	9.12 (6.46,11.78)^***^	7.49 (4.83,10.15)^***^	9.52 (6.85,12.20)^***^	2.03(− 0.66,4.73)
**LEQ**						
**Pain** T2	0.05(− 0.39,0.50)	− 0.55(− 1.0,-0.09)^*^	− 1.15(− 1.61,-0.69)^***^	−0.60(− 1.06,-0.14)^**^	−1.20(− 1.66,-0.74)^***^	−0.60(− 1.06,-0.13)^*^
T6	0.06(− 0.38,0.51)	0.15(− 0.29,0.61)	0.39(− 0.06,0.85)	0.09(− 0.36,0.55)	0.33(− 0.12,0.78)	0.23(− 0.22,0.70)
T12	0.09(− 0.36,0.53)	0.44(− 0.01,0.89)	0.96 (0.50,1.41)^***^	0.35(− 0.10,0.80)	0.87 (0.41,1.33)^***^	0.52 (0.06,0.98)
**Walk** T2	0.03 (− 0.2,0.26)	− 0.04(− 0.28,0.18)	0.15(− 0.09,0.38)	− 0.07(− 0.31,0.15)	0.11(− 0.11,0.35)	0.19(− 0.02,0.43)
T6	−0.01(− 0.23,0.22)	− 0.04(− 0.27,0.19)	0.20(− 0.03,0.43)	− 0.03(− 0.27,0.20)	0.20(− 0.03,0.44)	0.24 (0.01,0.48)^*^
T12	0.07(− 0.15,0.30)	0.08(− 0.14,0.32)	0.18(− 0.04,0.42)	0.01(− 0.22,0.24)	0.11(− 0.12,0.35)	0.10(− 0.14,0.34)
**ADL** T2	0.11(− 0.22,0.44)	− 0.01(− 0.34,0.32)	−0.29(− 0.62,0.04)	−0.12(− 0.45,0.21)	−0.40(− 0.73,-0.06)^*^	−0.27(− 0.61,0.06)
T6	0.03(− 0.29,0.36)	0.80 (0.46,1.13)^***^	1.02 (0.68,1.36)^***^	0.76 (0.43,1.10)^***^	0.98 (0.65,1.32)^***^	0.22(−0.12,0.56)
T12	0.05(−0.27,0.38)	0.65 (0.32,0.99)^***^	0.78 (0.44,1.11)^***^	0.59 (0.26,0.93)^***^	0.72 (0.38,1.06)^***^	0.12(−0.21,0.46)
**Total** T2	0.12(−0.59,0.84)	− 0.24(− 0.97,0.47)	− 1.29(− 2.02,-0.56)^***^	−0.36(− 1.09,0.35)	−1.42(− 2.15,-0.68)^***^	−1.05(− 1.79,-0.31)^**^
T6	0.07(− 0.64,0.78)	1.37 (0.65,2.10)^***^	1.61 (0.88,2.34)^***^	1.30 (0.57,2.03)^***^	1.54 (0.81,2.27)^***^	0.24(− 0.49,0.98)
T12	0.25(− 0.45,0.97)	1.63 (0.90,2.35)^***^	1.93 (1.20,2.65)^***^	1.37 (0.64,2.10)^***^	1.67 (0.93,2.40)^***^	0.29(−0.44,1.03)
**VAS (1–10)**						
T2	0.05 (−0.45,0.56)	−0.05 (− 0.56,0.45)	−0.72 (−1.24,-0.24)^**^	−0.11(− 0.62,0.40)	−0.78(− 1.30,-0.26)^**^	−0.67 (− 1.2,-0.14)
T6	0.04(− 0.46,0.55)	0.31(− 0.20,0.82)	0.57 (0.06,1.09) ^8^	−.26(− 0.25,0.77)	0.52 (0.01,1.04)^*^	0.26(− 0.25,0.78)
T12	−0.02(− 0.53,0.48)	0.75 (0.23,1.26)^**^	2.05 (1.53,2.56)^***^	0.77 (0.26,1.29)^**^	2.07 (1.56,2.59)^***^	1.29 (0.77,1.82)^***^

Abbreviations: *SD* standard deviation; *PRGF* plasma rich in growth factor; *PRP* platelet-rich plasma; *HA* hyaluronic acid; *VAS* visual analog scale; *WOMAC* Western Ontario and McMaster Universities Osteoarthritis Index; *LEQ* Lequesne Index; *MD* mean difference; *CI* confidences interval; *T2* 2nd month post injection, *T6* 6th month post injection, *T12* 12th month post injection

* Between-group effects *p* < 0.05; ** Between-group *p* < 0.01; *** Between-group *p* < 0.001

^a^ Adusted generalized estimating equations model after controlling the baseline Outcome, sex, age, BMI;

According to Tables 2 and 3 at the beginning of the study, no significant difference observed in the evaluated scores between groups (*P* > 0.05). In the 2 months post-injection evaluation, the ozone group had lower WOMAC, Lequesne, and VAS scores (better results) compared to other groups. The differences were significant in WOMAC (for Total score as well as Pain and Function sub-scores), and Lequesne (Total score and Pain sub-score). However, at the 6th month of follow up (Tables 2, 3, and Figs. 2, 3 and 4), patients treated with HA, PRP, PRGF demonstrated better results based on WOMAC, Lequesne, and VAS compared to those cases treated with ozone. At this stage, the WOMAC (Total, and Pain and Function sub-scores); Lequesne (Total and ADL sub-score) and VAS scores were observed significantly higher in ozone group than the other groups (*P* < 0.05).

In addition, in the 6th month of follow up, the VAS and WOMAC scores of the PRP and PRGF groups were lower than the HA group, however had somehow similar Lequesne scores. These differences though, were not found to be significant.

At the end of the 12th month (Tables 2, 3, and Figs. 2, 3 and 4), only PRGF and PRP groups had statistically significant differences from those treated with HA and ozone. The Total, Pain and Function scores of the WOMAC; the Total, Pain, and ADL scores of the Lequesne; and the VAS score were meaningfully lower in the PRGF and PRGF groups (P < 0.05) at the final timeline of this study. In the WOMAC Stiffness sub-score as well as in the Lequesne Walk sub-score, no significant differences were observed between the four groups 12 months after injection.

Of note, no significant variation was observed within the study groups for WOMAC, VAS and Lequesne scores.

As it is obvious in Figs. 2, 3 and 4, despite lower WOMAC, VAS, and Lequesne scores were observed at 2th month post-injection in all groups, these scores showed an increasing trend after the sixth months, which reaches its peak (near to the baseline) after 12 months. Although patients receiving ozone had the lowest scores 2 months after injection, they had a sharper increase in the later months and ended up with the highest scores among all groups.

The patients of the four groups were compared regarding their satisfaction and complications after injection. Accordingly, PRP and PRGF groups had experienced more but not significant post injection pain. Either there was no significant difference between four groups in patient’s satisfaction. (Tables 4, 5).

**Table 4:** Comparison of post injection adverse effects between four groups

	PRP	PGRF	HA	Ozone	P	Test
Post injection complications	0.32	0.41	0.24	0.43	0.56	Kruskal-Wallis

**Table 5:** The amount of satisfaction among the four groups

	PRP	PRGF	HA	Ozone	P Test
Very little	4 (7.7%)	5 (9.8%)	7 (14.3%)	8 (16.7%)	0.06 Kruskal-Wallis
Little	9 (17.3%)	8 (15.7%)	8 (16.3%)	7 (14.6%)	
Moderate	10 (19.2%)	9 (17.6%)	12 (24.5%)	19 (39.6%)	
Much	15 (28.8%)	17 (33.3%)	13 (25.6%)	9 (18.8%)	
Very much	14 (26.9%)	12 (23.5%)	9 (18.4%)	5 (10.4%)	

## Discussion

According to our study, in two months after injection, the patients of all four groups showed significantly lower scores in WOMAC, Lequesne, and VAS compared to their primary assessment before the injections (baseline levels). Based on the results, the ozone group had significantly lower WOMAC, Lequesne, and VAS scores than the other groups at 2th month of follow up, however its effects wiped out after 12 months. It is clear that the ozone therapy in knee OA has some early beneficial but not long lasting effects. In accordance with the results of present study, a previous meta-analysis performed by Raeissadat et al. showed that the ozone’s effects wear off 4–6 months post-injection [36]. Dernek et al. has also shown that compared to PRP, patients who treated with ozone have experienced earlier improvement in OA symptoms, but PRP had long term effects than ozone therapy [37]. Another study conducted by Gaballa et al. revealed that despite ozone being able to reduce the WOMAC score in similar amount as PRP at 1th month post-injection, but at 3th month of follow up, patient who received ozone therapy higher WOMAC scores [38]. Although the results obtained by Gaballa et al. was somehow similar to the findings of this study, but on the contrast to we found that PRP has much long-term effects. According to the literature, ozone therapy could increase the production of reactive oxygen species in the inflammatory site which can inactivate proteolytic enzymes and inhibit the release of proinflammatory cytokines, and therefore ameliorate the symptoms. However, over the short time the dissolved ozone might be cleared up from the synovial fluid leading to decreased therapeutic efficiencies [39]. Therefore, it seems that multiple doses of ozone might be beneficial and could be added to the other therapeutic regimens.

In our study, 6 months after injection, patients treated with HA, PRP, and PRGF showed better scores compared to ozone. The difference between HA, PRP, PRGF was not found to be statistically significant. Likewise, Raeissadat et al. has shown that HA and PRGF had similar effects 2 and 6 months after with no meaningful difference between the groups [12]. Furthermore, according to a study performed by Duymus et al., the effects of PRP, HA, and ozone were reported to be similar 1 month post injection; while 6 months after injection, PRP and HA were superior to ozone [40]. Despite the findings of the aforementioned study, results of another study performed in 2018 by Raeissadat et al. on 174 patients demonstrated no significant difference between HA and ozone 6 months after injection [30].

In our study, 12 months after injection, only patients who were treated with PRGF or PRP had meaningfully better results compared to those who had been treated with HA or ozone. In the study of Duymus et al. however, after 12 months, PRP had shown meaningfully better results than ozone and HA [40]. Superior effects have been reported for PRP compared to HA at 12 months post-injection [25, 35]. The better results of PRP compared to HA in 12 month follow up were also acknowledged in a meta-analysis by Wen-Li-Dai in 2016 [26]. The discrepancies between these studies might be due to the different methodologies or sample size used in these studies. However, mechanistically it has been proven that hyaluronate destruction occurs in the OA, thus although introduction of the exogenous HA could alleviate the symptoms and improve the functional impairment but cannot inhibit the inflammatory process in the knee OA [19]. Moreover, over the time the exogenous HA is destroyed in the inflammatory site and thus the symptoms start again after a period post-injection. In the case of the PRP or PRGF, it has been shown that these products could stimulate chondrogenesis, modulate the intra-articular microenvironment as well as cellular composition and proliferation, and directly affect the expression of some major inflammatory mediators in the joint, thus their effect may remain for a longer time compared to the ozone or HA [41].

Similarity in the effects of PRP and PRGF in 12-month follow up has been demonstrated in other research; for example, in 2012, Filardo et al. showed that 3 PRGF or PRP injections 3 weeks apart had no significant difference regarding improvements in pain and function of OA patients at 2, 6, and 12 month follow ups and both products had proven effective in this regard [42].

While comparing the results of different studies, different factors should be taken into consideration. Among the reasons for the diversity of results could be the differences in the PRP preparations used regarding platelet dosage (volume and concentration), purity (the existence of white and red blood cells and their concentration), efficacy of the product preparation based on the quality of the kit used as well as using or not using an activator. Different preparation methods and concentrations, despite having the same product name, could result in different products; which can in turn have different effects in changing a destructive articular environment into a regenerative one. In the case of HA, there could be differences in the volume, concentration, molecular weight, being linear or cross-linked, and the source (animal or fermentation). When considering ozone, variances in volume and concentration could result different therapeutic effects.

Among other reasons causing variations in results can be the differences in the number of injections and the time intervals between them. In various studies, a range of one to several injections has been performed, which have been spaced between 1 week to 3 or 4 weeks apart. As can be observed in the different studies, no agreement exists upon a standard frequency or number of injections [9, 43]. Therefore, based on our own previous experience with plasma-based products and in order to reach a balance between groups regarding the cost of treatment, we chose 2 injections with 3 weeks separation for PRP and PRGF; while 3 weekly injections were considered for HA and ozone. Other possible reasons for discrepancies in results may stem from the variety in rehabilitative protocols employed after injections as well as the ways in which assessment of response to treatment is performed. Demographic differences (age or gender), amount of activity, and severity of osteoarthritis also play a role in the results.

### Limitations and strengths

One of the limitations of this study is the lack of a placebo group. In addition, due to their nature, some aspects of the study were also not blinded; PRP and PRGF required blood samples from the candidates as well as a specific injection program. Taking blood samples from HA and ozone candidates would not have been ethically approved. All other aspects of the study such as data analysis and follow up remained blinded.

The concurrent comparison of four different novel treatment methods for knee OA can be considered as one of the strengths of this study. To the best of our knowledge, at the time of this research, no study has compared all four of these at the same time. The long patient follow up time of 12 months is also another strength of our work.

## Conclusions

With the results of the current study in mind, although ozone may yield satisfactory short-term results compared to HA, PRP, and PRGF; It is PRP and PRGF which can improve symptoms of knee OA in the long run compared to HA and ozone. Therefore, these products seem to be the preferable choices for long-term management; especially since according to a study by Stefano Landi in 2018, the use of PRP compared to HA does not only yield better results, but is also more cost effective [44].

## Data Availability

The datasets used and/or analyzed during the current study are available from the corresponding author on reasonable request.

## Abbreviations

PRP: Platelet Rich Plasma
PRGF: Plasma Rich in Growth Factor
HA: Hyaluronic Acid
IAIs: Intra Articular Injections
OA: Osteoarthritis
WOMAC: Western Ontario and McMaster Universities Osteoarthritis Index
PDGF: Platelet Derived Growth Factor
IGF: Insulin-like Growth Factor
CBC: Complete Blood Count
CRP: C-reactive protein
ESR: erythrocyte sedimentation rate
AP: anteroposterior
VAS: Visual Analogue Scale
ADL: Activities of Daily Life
